# The Autosomal Recessive Inheritance of Hereditary Gingival Fibromatosis

**DOI:** 10.1155/2013/432864

**Published:** 2013-12-14

**Authors:** Poulami Majumder, Vineet Nair, Malancha Mukherjee, Sujoy Ghosh, Subrata Kumar Dey

**Affiliations:** ^1^Department of Biotechnology, Centre for Genetic Studies, School of Biotechnology and Biological Sciences, West Bengal University of Technology, BF-142, Sector I, Salt Lake City, Kolkata, West Bengal 700064, India; ^2^Department of Periodontics & Oral Implantology, Dr. R. Ahmed Dental College & Hospital, Kolkata, West Bengal 700014, India; ^3^Department of Zoology, University of Calcutta, 35 Ballygunge Circular Road, Kolkata, West Bengal 700019, India

## Abstract

Hereditary gingival fibromatosis (HGF) is a rare condition which is marked by enlargement of gingival tissue that covers teeth to various extents leading to aesthetic disfigurement. This study presents a case of a 28-year-old female patient and 18-year-old male who belong to the same family suffering from HGF with chief complaint of overgrowing swelling gingiva. The presence of enlarged gingiva with the same eruption was found in their other family members with no concomitant drug or medical history, and the occurrence of HGF has been found in one generation of this family which may indicate the autosomal recessive inheritance pattern of HGF. Hereditary gingival fibromatosis is an idiopathic condition as its etiology is unknown and it was found to recur in some cases even after surgical treatment. Both patients underwent thorough oral prophylaxis and later surgical therapy to correct the deformity.

## 1. Introduction

Hereditary gingival fibromatosis (HGF) is a clinical condition defined as an overgrowth of gingiva [1]. This is an important and very rare dental case (1 in 750,000) which is also referred to as idiopathic gingival hyperplasia in medical history [1, 2]. The maxillary and mandibular tissues of both arches are affected by slow enlargement of gingiva. This is considered as a benign condition, that is, associated with various factors like inflammation, hormonal imbalance, neoplasia, and some unknown causes [3, 4]. Healthy gingival tissue is characterized as pink, firm, fleshy, and made up of connective tissue covered by mucous membrane surrounding the neck of the teeth in a collar-like fashion on the jaw. However the HGF present in most of the anatomic crown of teeth causes abnormally shaped teeth, abnormal tooth movement, speech disorders, and other dental and oral problems [5].

It has been reported that both males and females are equally affected, with the phenotype and genotype frequency of HGF being 1 : 175,000 and 1 : 350,000, respectively [4]. HGF is more commonly associated with an autosomal dominant gene [5, 6]. According to various studies the pedigree analyses of HGF families confirm the autosomal dominant inheritance of HGF trait, although autosomal recessive or even as X-linked inherited cases have also been reported in some literature [7, 8]. Mutation in SOS-1 or son-of-sevenless gene is responsible for this disease; this has been reported by several authors [5, 9–11]. It is a guanine nucleotide-exchange factor that functions in the transduction of signals that control cell growth and differentiation [11]. Chromosomes numbers 2, 4, and 5 are found with their specific genetic loci including 2p21-p22 (GINGF), 2p13-p16, 2p22.3-23 (GINGF3), 5q13-q22 (GINGF2), 4q21, and 4q in association with HGF that enables mutations, duplications, deletions, and other genetic anomalies to take place. Other genetic loci like 8, 14q, 19p, 19q, and Xq are also related to various syndromes associated with hereditary gingival fibromatosis [11]. In current literatures it has been reported that a mutation in the son-of-sevenless-1 (SOS1) gene, which results in a single nucleotide insertion, causes hereditary gingival fibromatosis [11].

## 2. Case Presentation

There were two patients of 28 years and 18 years who were reported from Dr. R. Ahmed Dental College & Hospital, Kolkata, West Bengal, with the chief complaint of overgrowing gingiva surrounding all of their teeth. Those patients were referred for chromosomal diagnosis at our laboratory in Centre for Genetic Studies, West Bengal University of Technology.

### 2.1. Family History

The family history of affected persons was determined by questioning of the index case and it was confirmed that there were other family members who also had the symptoms of gingival overgrowth and that was subsequently confirmed by clinical examination of most of them. Among them two were females and the rest were male and they were siblings of the same parents.

**Figure 1:**
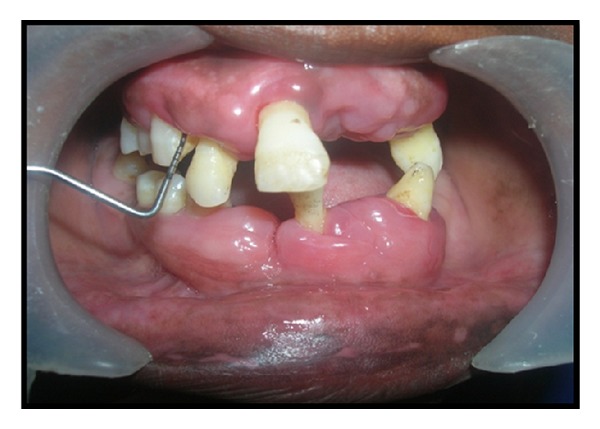
Female patient with gingival enlargement (preoperative view).

**Figure 2:**
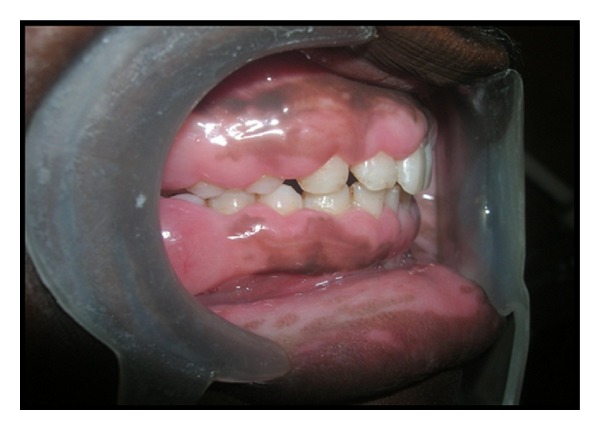
Male patient with gingival enlargement in milder form (preoperative view).

**Figure 3:**
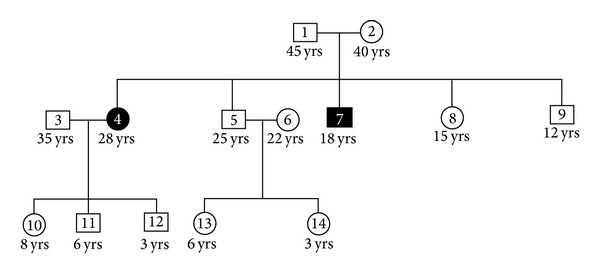
Pedigree of patient's family.

A 28-year-old female patient was reported to the Department of Periodontics & Oral Implantology, Dr. R. Ahmed Dental College & Hospital, with the chief complaint of swelling in the gingiva for four years (Figure 1). Her 18-year-old brother was also reported with the same complaint but in milder form (Figure 2). According to the information provided by index cases, swelling of gingiva arose on approximately 3 to 4 years earlier and caused difficulties in speaking and eating but they had no history of oral pain. The female patient was married for 10 years and she had three children within the age range of 3–8 years and none of them were affected. The other members of their family like their parents and their other two brothers were unaffected. The family history of three generations was available and it was understood that the trait of gingival fibromatosis followed autosomal recessive pattern of inheritance in this family (Figure 3).

### 2.2. Medical History

Patients' medical history was normal. There was no history of taking long-term medicine for any particular disease. There was also no record of mental retardation or hypertrichosis and no other sign of clinical symptoms that could be associated with gingival enlargement.

### 2.3. Clinical Diagnosis

This enlargement affects the attached gingiva, as well as the gingival margin and interdental papilla, in contrast to phenytoin induced enlargement, which is often limited to the gingival margin and interdental papillae. The facial and lingual surfaces of the mandible and maxilla are generally affected, but the involvement may be limited to either jaw. The enlarged gingiva is pink, firm, and almost leathery in consistency and has a characteristic minutely pebbled surface. In severe cases teeth are almost completely covered, and the enlargement projects into the oral vestibule. The jaws appear distorted because of the bulbous enlargement of the gingiva. Secondary inflammatory changes are common at the marginal gingiva.

## 3. Treatment and Surgical Procedure

**Figure 4:**
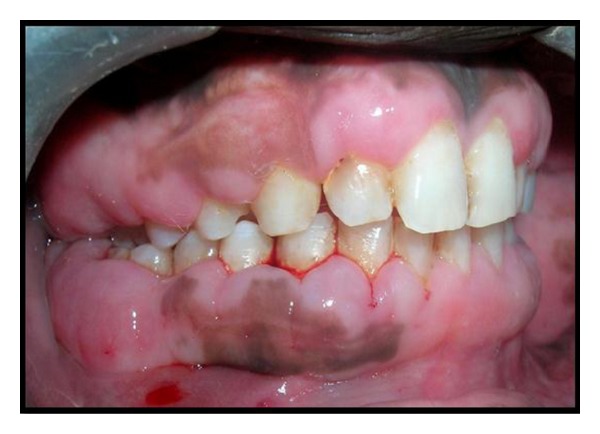
Pocket marker marked the bleeding points that denoted the excess portions of gum of male patient.

**Figure 5:**
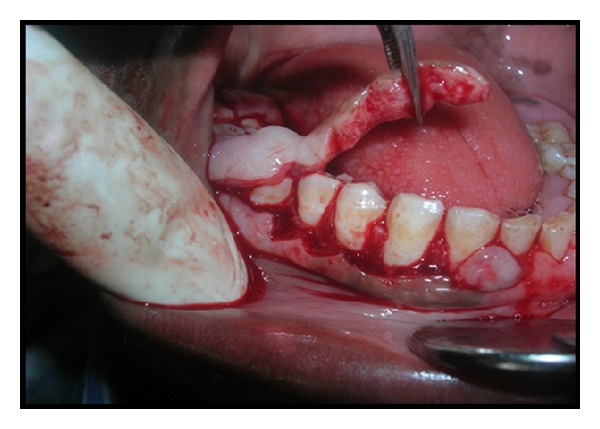
Internal bevel gingivectomy was done to remove the excess tissue of the male patient.

**Figure 6:**
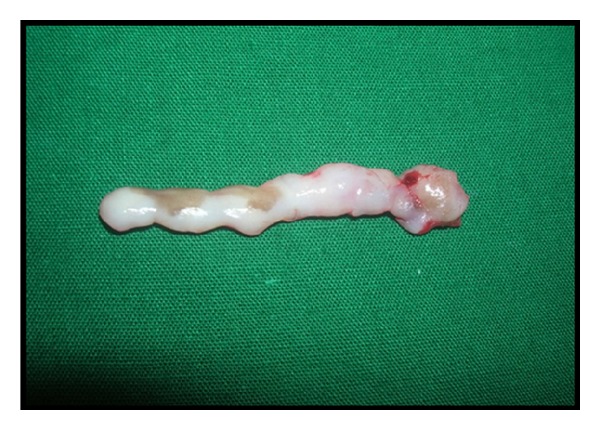
Surgically removed excess tissue.

**Figure 7:**
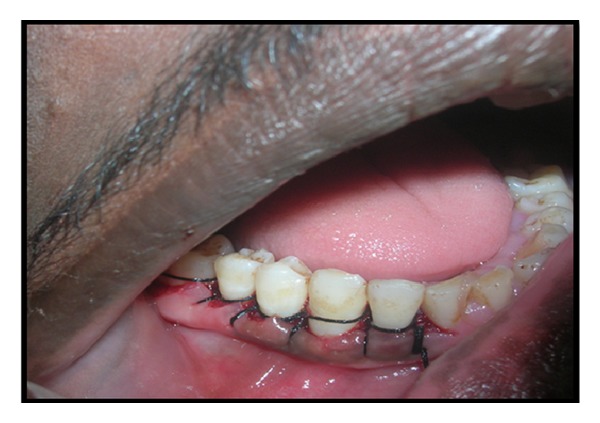
Gingivoplasty was done and continuous sling sutures were placed.

**Figure 8:**
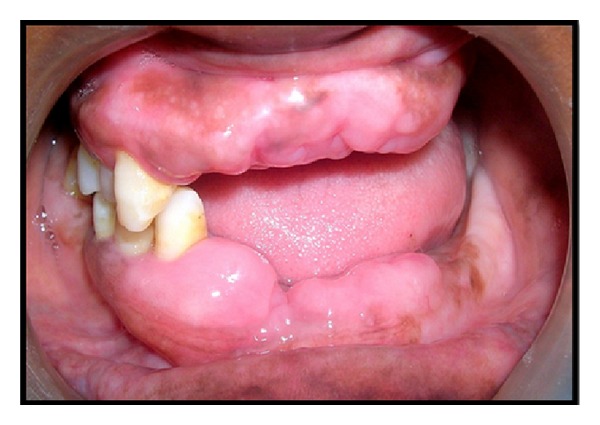
Postoperative view of female patient. It was shown that the left side gingival enlargement was cured.

**Figure 9:**
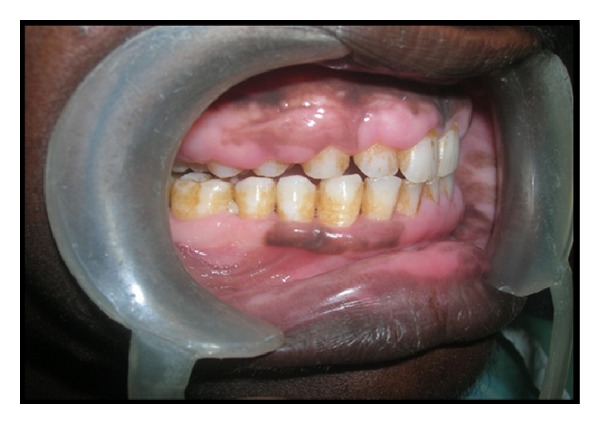
Postoperative view of male patient after six months.

There is no such particular treatment or medicine for complete prevention of gingival fibromatosis but surgical procedure may cure this problem to some extent provided oral hygiene is thoroughly maintained. After local anesthesia bleeding points were marked on the gums of patients with the help of pocket marker (Figure 4). The excess tissue was removed by performing an internal bevel gingivectomy or undisplaced flap (Figures 5 and 6). Gingivoplasty was performed and continuous sling sutures were placed and periodontal dressing was applied (Figure 7). After 7 days sutures were removed and after 4 weeks they were recalled for further evaluation (Figure 8). Reevaluation was done after six months but no recurrence was observed (Figure 9). As the female patient was heavily affected, the ratio of cure was less than her brothers. Her left side gingival enlargement was cured but the right side is under observation.

## 4. Discussion

Here, we report a rare case of hereditary gingival fibromatosis which was not found in the last four years in Kolkata and is transmitted as an autosomal recessive inheritance. In this family, another female sibling who is 15 years old possesses similar clinical features which we may consider as the early symptoms of gingival fibromatosis. So it has been noticed that there is a tendency of sudden appearance of swelling gums around teeth at around 15 to 16 years of age, although some other studies report the occurrence of HGF also found in early age as well as juvenile state [12, 13]. Consequent to this there is compromised chewing efficiency and increasing mobility of teeth which may eventually lead to loss of teeth. The patients reported one month after they had undergone the surgical procedure and were again reviewed six months later. They were found to be in a stable condition though recurrence of HGF cannot be ruled out in the future. Generally surgical intervention after scaling and root planing of teeth and sufficient oral homecare is sufficient to alleviate the symptoms [3, 4]. In some literatures, various associated disorders of gingival enlargement had been discussed that include Zimmermann Laband syndrome (ear, nose, bone, and nail defects with hepatosplenomegaly), Rutherford syndrome (oculodental syndrome), Jones syndrome (progressive deafness), Cross syndrome (microphthalmia, mental retardation, athetosis, and hypopigmentation), Murray-Puretic Drescher syndrome (juvenile hyaline fibromas), and Ramon syndrome reasons, [9, 14–16], that is, why HGF is considered as part and parcel of multisystem syndrome [2, 10]. However, in this case, a thorough evaluation of the patients revealed that there was no association of HGF with any of the above syndromes.

## 5. Conclusion

Hereditary gingival fibromatosis stands apart from other gingival enlargements in the varied treatment options available and the nature of recurrence posttreatment. There is no consensus among authors related to the mode of treatment. Here, in this present case report, we highlight the autosomal inheritance patterns and the surgical treatment found in HGF cases.
